# Sarcomatoid hepatocellular carcinoma mimics hepatic abscess on contrast‐enhanced ultrasound

**DOI:** 10.1002/ccr3.8456

**Published:** 2024-01-29

**Authors:** Haibo Luo, Xiaoling Leng, Guiwu Chen, Wenqin Liu, Yanhua Xie

**Affiliations:** ^1^ Department of Ultrasound The Tenth Affiliated Hospital of Southern Medical University, Dongguan People's Hospital Dongguan Guangdong China

**Keywords:** computer tomography, hepatocellular carcinoma, Sarcomatoid, ultrasonography

## Abstract

Sarcomatoid hepatocellular carcinoma (SHCC) is a rare subtype of hepatocellular carcinoma characterized by abdominal pain or persistent fever with an inflammatory reaction. Here, we report a case of SHCC mimicking hepatic abscess described by not only ultrasonography but also computer tomography. SHCC is a rare subtype of hepatocellular carcinoma characterized by epithelial and mesenchymal tumor features with sarcomatoid morphology. Here, we report a case of SHCC described by ultrasonography and computer tomography as well as confirmed by pathological examination.

## Graphical abstract

A 71‐year‐old male patient with a mass located in the liver was described by imaging, including routine abdominal ultrasound, contrast‐enhanced ultrasound, plain computer tomography, and enhanced computer tomography. A diagnosis of sarcomatoid hepatocellular carcinoma (SHCC) was confirmed by pathological examination.

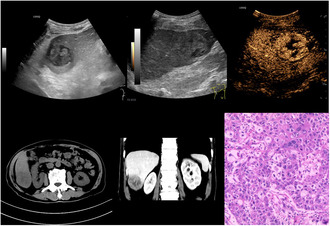

Sarcomatoid hepatocellular carcinoma (SHCC), is a rare subtype of hepatocellular carcinoma characterized by epithelial and mesenchymal tumor features with sarcomatoid morphology, whose incidence is only 5% of hepatocellular carcinoma.^1^ Compared with hepatocellular carcinoma, SHCC has more rapid tumor growth and poorer disease progression. However, SHCC is easily misdiagnosed and delays the optimal treatment due to the clinical and imaging features being similar to hepatic abscesses. Here, we present a case image, including routine abdominal ultrasound, contrast‐enhanced ultrasound, plain computer tomography, and enhanced computer tomography, of SHCC that mimics hepatic abscesses.

A 71‐year‐old male patient presented to our hospital with a fever of unknown origin for 1 month, whose temperature is approximately 38°C. Physical examination revealed a right epigastrium slight tenderness. Laboratory tests revealed hypersensitive C‐reactive protein of >5.00 mg/L, routine C‐reactive protein of >200.00 mg/L, white blood cell of 21.36 × 10^9^ /L, neutrophils of 18.43 × 10^9^ /L, activated partial thromboplastin time of 45.30 s, fibrinogen quantification of 8.21 g/L, hepatitis B virus surface antigen of <0.01 IU/mL, hepatitis B virus surface antigen–antibody of 32.12 mIU/mL, hepatitis B virus e antigen of 0.439 S/CO, hepatitis B virus e antigen–antibody of 1.59 S/CO, and hepatitis B virus core antigen–antibody of 6.41 S/CO, alpha feto protein of 50.98 ng/mL.

Initially, routine abdominal ultrasound explored a hypoechoic mass located in the right hepatic lobe with irregular anechoic areas internally (Figure 1). Additionally, a contrast‐enhanced ultrasound indicated a mass with rapidly high peripheral enhancement during the arterial phase and with low peripheral enhancement during the portal venous phase without internal enhancement throughout all the phases (Figure 2). Furthermore, the mass had low density with internal cystic degeneration on plain computer tomography and obvious peripheral enhancement with no internal enhancement on enhanced computer tomography (Figure 3). Ultimately, the patient underwent a surgical mass excision, and an SHCC diagnosis was confirmed (Figure 4). No recurrence or metastasis was found during the 5‐month postoperative follow‐up.

**FIGURE 1:**
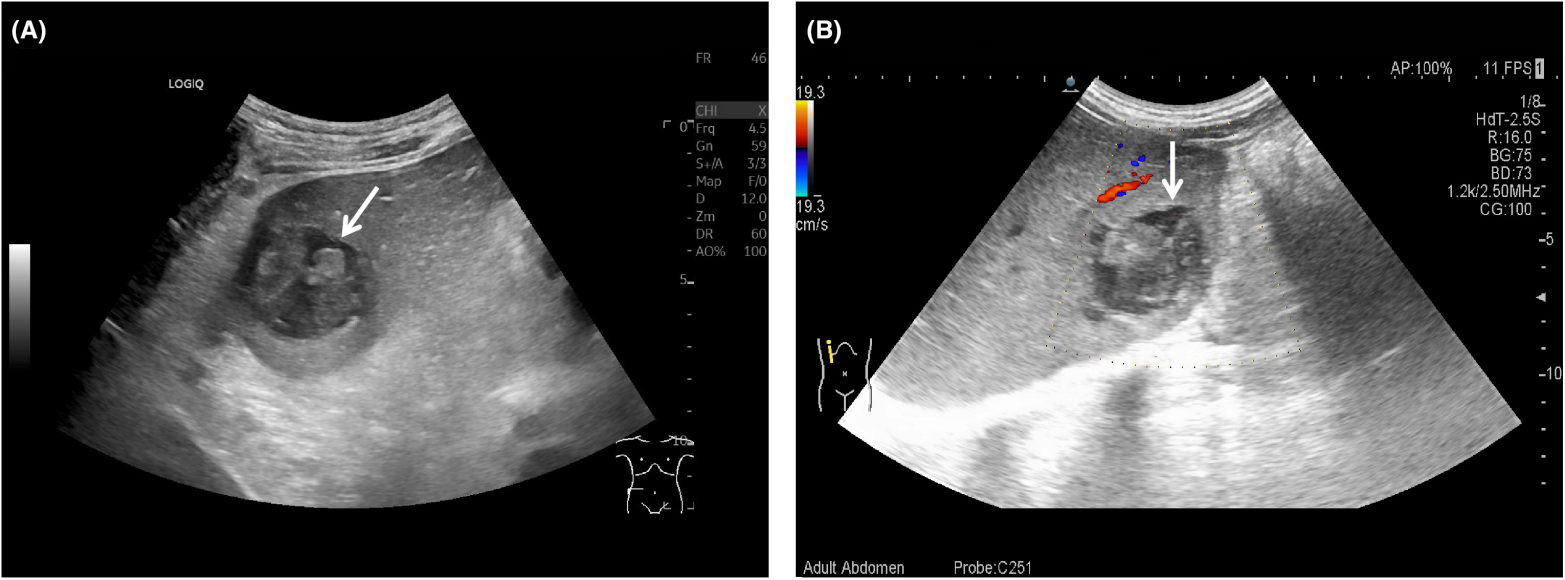
Routine abdominal ultrasound of sarcomatoid hepatocellular carcinoma. (A) Grayscale ultrasound showed a cystic‐solid mass (arrow) located in the right hepatic lobe that was irregular, ill‐defined, inhomogeneous, and approximately 56 × 53 × 44 mm in size. (B) Color Doppler flow imaging showed no blood flow signal inside the mass (arrow).

**FIGURE 2:**
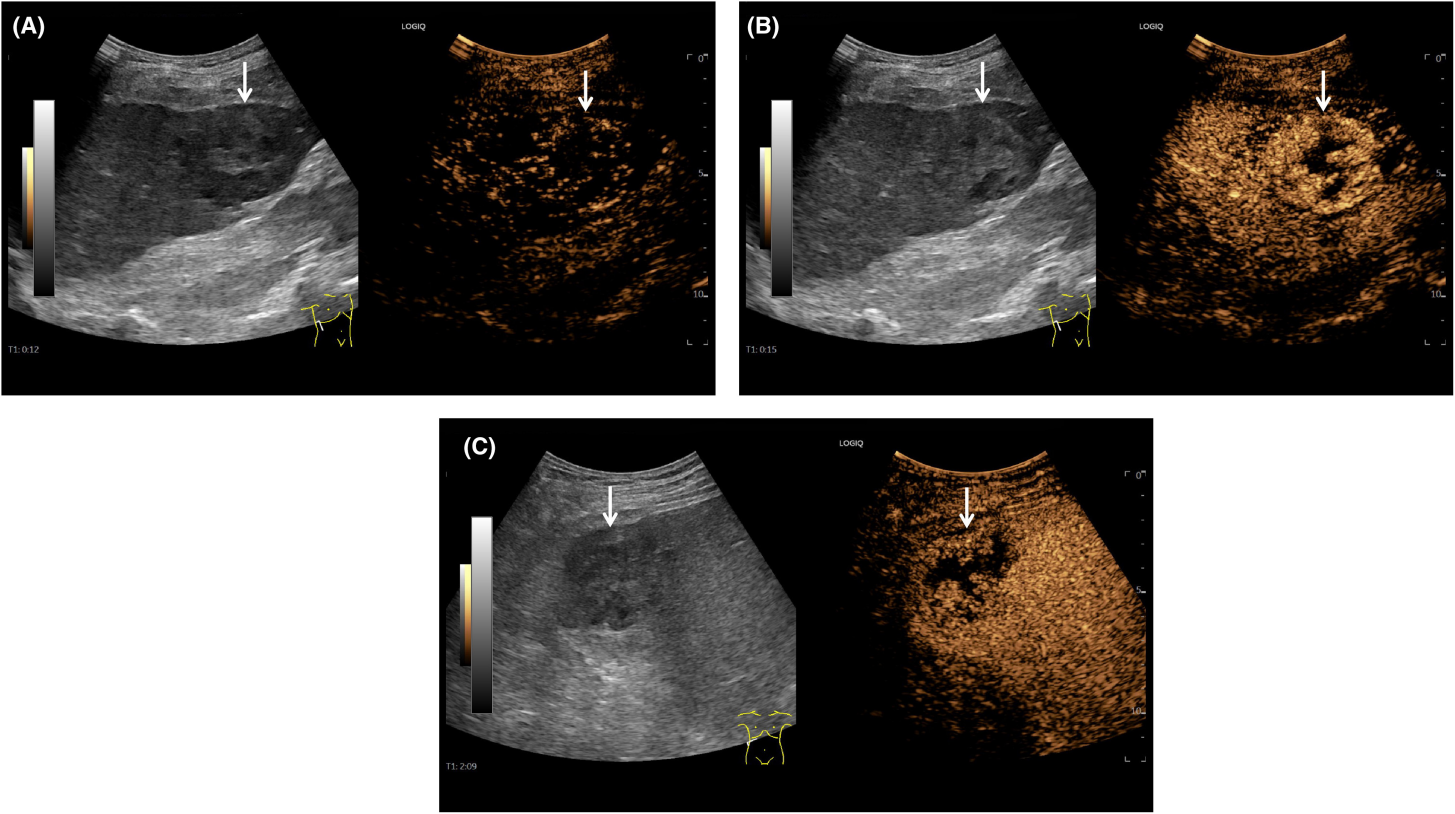
Contrast‐enhanced ultrasound of sarcomatoid hepatocellular carcinoma. (A) In the early arterial phase, the mass (arrow) rapidly enhanced beginning in the 12th second. (B) In the arterial phase, the mass (arrow) was high peripheral enhancement with no internal enhancement in the 15th second. (C) In the portal phase, the mass (arrow) was washed out in the 129th second.

**FIGURE 3:**
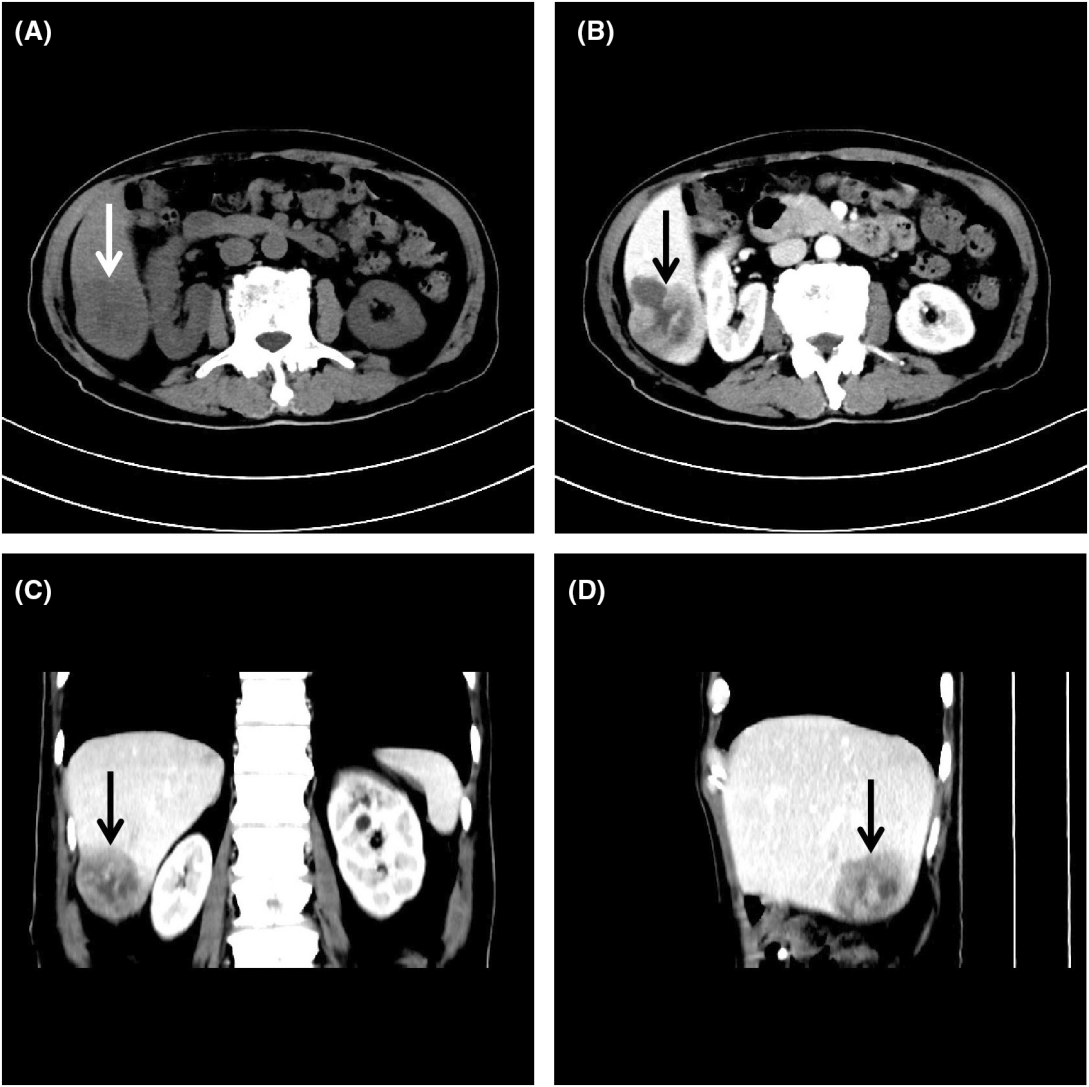
Computer tomography of sarcomatoid hepatocellular carcinoma. (A) Plain computer tomography showed the mass (arrow) was low‐density, ill‐defined, and approximately 60 × 47 × 50 mm in size with a value of 32 to 36 HU. (B–D) Enhanced computer tomography showed the mass (arrow) was obviously peripheral enhancement with a value of 63 to 84 HU while no enhancement inside.

**FIGURE 4:**
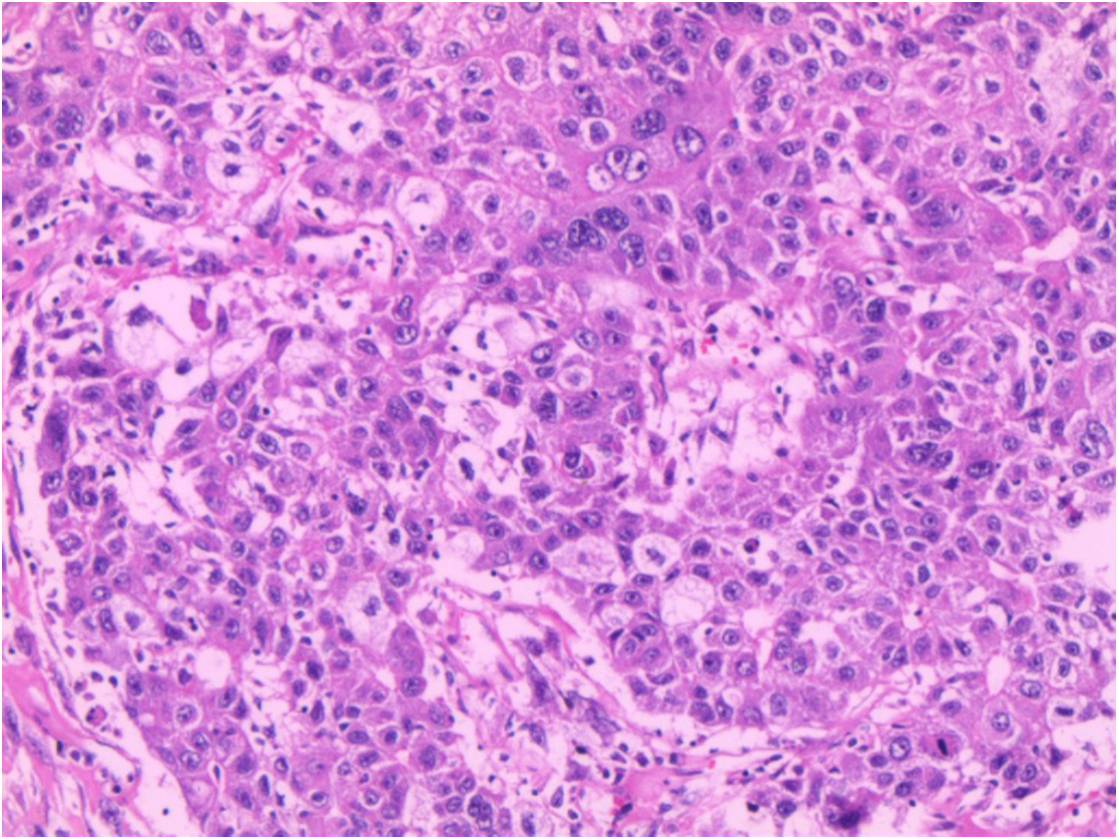
Pathology of sarcomatoid hepatocellular carcinoma. Hematoxylin–eosin staining showed the cells of the tumor were proliferating, heterotypic, glandular, and diffusely patchy in shape, whose cytoplasm was abundant as well as nuclei were enlarged and strangely.

According to the previous literature, the patient of SHCC has abdominal pain or persistent fever with an inflammatory reaction due to infection and necrosis in malignancy. Routine abdominal ultrasound and plain computer tomography reveal the mass is diffuse liquefactive necrosis with or without metastasis or portal and hepatic vein thrombosis.^2^ Contrast‐enhanced ultrasound and enhanced computer tomography observe the mass with irregular, thick, circular, and high enhancement.^3^ However, the evidence of SHCC remains too deficient to differentiate it from hepatic abscesses, and thus further research is necessary.

## AUTHOR CONTRIBUTIONS

**Haibo Luo:** Writing – original draft. **Xiaoling Leng:** Supervision. **Guiwu Chen:** Writing – review and editing. **Wenqin Liu:** Formal analysis; visualization. **Yanhua Xie:** Formal analysis; visualization.

## FUNDING INFORMATION

No funding was received for this study.

## CONFLICT OF INTEREST STATEMENT

The authors declare no conflicts of interest.

## CONSENT

Written informed consent was obtained from the patient to publish this report in accordance with the journal's patient consent policy.

## Data Availability

The data used to support the findings of this study are available from the corresponding author upon request.
